# The effects of birth weight and estimated breeding value for protein deposition on nitrogen efficiency in growing pigs

**DOI:** 10.1093/jas/skab101

**Published:** 2021-03-29

**Authors:** Carola M C Van der Peet-Schwering, Lisanne M G Verschuren, Rob Bergsma, Mette S Hedemann, Gisabeth P Binnendijk, Alfons J M Jansman

**Affiliations:** 1 Wageningen Livestock Research, Wageningen, The Netherlands; 2 Topigs Norsvin Research Center B.V., Beuningen, The Netherlands; 3 Agrocampus Ouest, UMR1348 Pegase, Saint-Gilles, France; 4 Department of Animal Science, Aarhus University, Foulum, Denmark

**Keywords:** birth weight, estimated breeding value for protein deposition, growing pigs, nitrogen efficiency, nitrogen retention

## Abstract

The effects of birth weight (**BiW**; low BiW [**LBW**] vs. high BiW [**HBW**]) and estimated breeding value (**EBV**) for protein deposition (low EBV [**LBV]** vs. high EBV [**HBV**]) on N retention, N efficiency, and concentrations of metabolites in plasma and urine related to N efficiency in growing pigs were studied. At an age of 14 wk, 10 LBW–LBV (BiW: 1.07 ± 0.09 [SD] kg; EBV: −2.52 ± 3.97 g/d, compared with an average crossbred pig with a protein deposition of 165 g/d), 10 LBW–HBV (BiW: 1.02 ± 0.13 kg; EBV: 10.47 ± 4.26 g/d), 10 HBW–LBV (BiW: 1.80 ± 0.13 kg; EBV: −2.15 ± 2.28 g/d), and 10 HBW–HBV (BiW: 1.80 ± 0.15 kg; EBV: 11.18 ± 3.68 g/d) male growing pigs were allotted to the experiment. The pigs were individually housed in metabolism cages and were subjected to an N balance study in two sequential periods of 5 d, after an 11-d dietary adaptation period. Pigs were assigned to a protein adequate (**A**) or protein restricted (**R**, 70% of A) regime in a change-over design. Pigs were fed 2.8 times the energy requirements for maintenance. Nontargeted metabolomics analyses were performed in urine and blood plasma samples. The N retention (in g/d) was higher in the HBW than in the LBW pigs (*P* < 0.001). The N retention (in g/[kg metabolic body weight (BW^0.75^) · d]) and N efficiency, however, were not affected by the BiW of the pigs. The N retention (*P* = 0.04) and N efficiency (*P* = 0.04) were higher in HBV than in LVB pigs on the A regime but were not affected by EBV in pigs on the R regime. Restricting the dietary protein supply with 30% decreased the N retention (*P* < 0.001) but increased the N efficiency (*P* = 0.003). Nontargeted metabolomics showed that a hexose, free amino acids (**AA**), and lysophosphatidylcholines were the most important metabolites in plasma for the discrimination between HBV and LBV pigs, whereas metabolites of microbial origin contributed to the discrimination between HBV and LBV pigs in urine. This study shows that BiW does not affect N efficiency in the later life of pigs. Nitrogen efficiency and N retention were higher in HBV than in LBV pigs on the A regime but similar in HBV and LBV pigs on the R regime. In precision feeding concepts aiming to further optimize protein and AA efficiency in pigs, the variation in EBV for protein deposition of pigs should be considered as a factor determining N retention, growth performance, and N efficiency.

## Introduction

Optimizing nitrogen (N) efficiency is essential to increase the sustainability of pig production systems (FAO, 2006) and to reduce the environmental impact. Several studies have indicated that N efficiency can be improved by more precisely adjusting protein and amino acids (**AA**) supply via the diet to the AA requirements of individual pigs (Ferket et al., 2002; Pomar et al., 2014). The AA requirements for maximal protein deposition depend on many factors including phenotypic (e.g., birth weight [**BiW**]) and genetic factors (e.g., estimated breeding value [**EBV**] for protein deposition) in individual pigs. The development of hyper-prolific sows has increased not only the number of piglets born per sow per year but also the number of piglets with a low birth weight (**LBW**; Quiniou et al., 2002). LBW piglets have a lower number of muscle fibers at birth (Alvarenga et al., 2013) and eat and grow less during the weaning and growing period (Rehfeldt et al., 2008; Alvarenga et al., 2013) compared with high birth weight (**HBW**) piglets. This may result in differences in N retention between LBW and HBW pigs in the growing phase. Van der Peet-Schwering et al. (2020) showed that N retention in g/(kg metabolic body weight [**BW**^**0.75**^] · d) and N efficiency in growing male pigs were not affected by BiW. The N retention in g/d, however, was higher in the HBW than in the LBW pigs. Moreover, HBW pigs showed lower plasma concentrations of α-amino N and metabolites derived from AA, suggesting a higher protein deposition in these pigs.

The development of DNA sequencing techniques to evaluate the genome variability allows to predict the EBV of individual pigs. Pigs with a high EBV (**HBV**) may have a higher N efficiency than pigs with a low EBV (**LBV**). Quantitative information about the effects of EBV on N efficiency and plasma metabolites related to N efficiency in growing pigs, however, is lacking.

Therefore, in this experiment, the effects were determined of both BiW and EBV in dependence of dietary protein supply on between animal variation in N retention, N efficiency, and concentrations of metabolites in plasma and urine related to N metabolism in growing pigs of 14 wk of age.

## Material and Methods

All procedures applied were in agreement with the Dutch law on animal experiments and approved by the Animal Ethical Committee of Wageningen Livestock Research.

### Animals, genotyping of animals, housing, and design

At an age of 14 wk, 10 LBW–LBV (BiW: 1.07 ± 0.09 [SD] kg; EBV: −2.52 ± 3.97 g/d, compared with an average crossbred pig with a protein deposition of 165 g/d), 10 LBW–HBV (BiW: 1.02 ± 0.13 kg; EBV: 10.47 ± 4.26 g/d), 10 HBW–LBV (BiW: 1.80 ± 0.13 kg; EBV: −2.15 ± 2.28 g/d), and 10 HBW–HBV (BiW: 1.80 ± 0.15 kg; EBV: 11.18 ± 3.68 g/d) male growing pigs (Synthetic boar × [Dutch Landrace × Large White]) were allotted to the experiment. The difference in EBV between LBV and HBV pigs was 13.2 g/d (−2.34 vs. 10.83 g/d). This means that HBV pigs have a 13.2 g/d higher genetic protein deposition capacity than LBV pigs. The pigs were born at the Swine Innovation Centre Sterksel, the Netherlands, and were selected from 17 litters. The LBW pigs had a BiW of 1.30 kg or lower, and the HBW pigs had a BiW of 1.55 kg or higher, similar to those used by Van der Peet-Schwering et al. (2020). Within 24 h after birth, a tissue sample was taken from the ear of the piglets while inserting an identity tag (Eartag FlexoPlus P Geno) and put into a test tube marked with a bar code for identification. The EBV of individual pigs was derived from genotyping the individual pigs with a 50K single-nucleotide polymorphism chip and genomic prediction of the protein deposition capacity by Topigs Norsvin, Beuningen, the Netherlands. For calculating EBVs, data of about 1.9 * 10^6^ purebred and 0.6 * 10^6^ (F2) crossbred animals collected over the past 6 yr were available. For all animals, at least live time daily BW gain was available. The trait with the lowest number of observations, average daily feed intake, still contained data on 170,000 purebred and 19,000 crossbred animals. The pedigree consisted of about 2.8 * 10^6^ animals of which 340,000 were genotyped (320,000 purebreds and 20,000 crossbreds). The genotyped animals were used to create a genomic relationship matrix applying the so-called algorithm for proven and young animals (Misztal, 2016). Both the traditional relationship matrix based on the pedigree and the genomic relationship matrix were blended to create a joint relationship matrix, the so-called H-1. The latter is used for breeding value estimations in pigs. Breeding values were estimated using MiXBLUP (Ten Napel et al., 2018). The genotyped pigs in the present study were added to the pedigree database so that EBV could be calculated for these pigs.

Piglets were weaned at an age of 28 d (range 26 to 30 d) and moved to the rooms for growing and finishing (**GF**) pigs at an age of 63 d. From weaning till the age of 98 d, LBW and HBW pigs were housed in separate pens (12 animals per pen) in the same rooms and were fed ad libitum. The LBW and HBW pigs were fed the following diets from weaning at day 28 till day 98: a weaner diet (net energy [**NE**]: 9.77 MJ/kg; crude protein: 160 g/kg) for 2 wk, a piglet diet (NE: 9.94 MJ/kg; crude protein: 163 g/kg) for 3 wk, and then a starter diet (NE: 9.86 MJ/kg; crude protein: 165 g/kg) for 5 wk. At day 98, the LBW–LBV, LBW–HBV, HBW–LBV, and HBW–HBV pigs, weighing on average 48.3 ± 3.9, 49.7 ± 4.1, 60.3 ± 4.7, and 59.5 ± 3.6 kg, respectively, were transported to the research facility “Carus” of Wageningen University, the Netherlands, and individually housed in metabolism cages (1.80 × 0.80 m) at a room temperature of 22 °C. They were subjected to N balance measurements in two sequential periods of 5 d using a restricted feeding regime. After a 6-d adaptation period to the metabolism cages, during which the pigs were fed a starter diet (NE: 9.86 MJ/kg; crude protein: 165 g/kg), pigs were adapted for 5 d to the experimental diets before the start of the first 5-d balance period. Pigs were assigned to a protein adequate (**A**) or protein restricted (**R**, 70% of A) regime in a change-over design. Five randomly selected pigs of each of the LBW–LBV, LBW–HBV, HBW–LBV, and HBW–HBV groups (in total 20 pigs) were allotted to the dietary regime A in balance period 1. The other 20 pigs were allotted to the dietary regime R in balance period 1. Between the two balance periods, there was an adaptation period of 5 d to the changed-over dietary treatment. Temperature and ventilation rate in the room were automatically controlled and appropriate for the growing stage of the pigs. The room was illuminated with artificial light from 0700 to 1900 hours.

### Diets and feeding

From 5 d before the first balance period, pigs received daily an A or R (70% of A) amount of dietary protein while providing the same amount of other nutrients. Therefore, during diet manufacturing, a basal mixture of protein-free ingredients was prepared. The diet for the A regime was composed of the basal mixture, which was supplemented with the protein sources casein, wheat gluten meal, and potato protein, and the free AA l-Lysine HCl and l-Threonine in the intended amounts. The diet for the A regime met the requirements for essential AA for growing pigs in the range of 40 to 70 kg BW (CVB, 2012). The diet for the R regime included the basal mixture to which 70% of the quantity of the protein sources (casein, wheat gluten meal, potato protein, l-Lysine HCl, and l-Threonine) was added compared with the inclusion in the diet for the A regime. The apparent ileal digestible methionine + cystine: lysine, threonine: lysine, and tryptophan: lysine ratios in both the A and R diets were 60%, 65%, and 19%, respectively. In order to supply all pigs with the same amount of protein-free ingredients, relative to their BW^0.75^, the feed allowance of pigs assigned to the R regime was 94.4% of that of pigs receiving the A regime. Titanium dioxide (0.4%) was included in the experimental diets as an indigestible marker. The ingredient and nutrient composition of the diets is presented in Table 1. The experimental diets were provided in mash form, mixed with water using a feed to water ratio of 1: 2, and provided to the pigs as a liquid feed. The diets were supplied as a liquid feed to stimulate the intake of the meals provided. Pigs were fed at 0800 and 1530 hours in equal amounts at 2.8 times the metabolizable energy (**ME**) requirements for maintenance (458 kJ ME/[kg BW^0.75^ · d]; ARC, 1981). Feed allowance during balance period 1 was based on the BW of each individual pig on day 104 and the expected daily gain from day 104 to 114. Feed allowance during balance period 2 was based on the BW of each individual pig on day 114 and the expected daily gain from day 114 to 125. Feed refusals were removed and weighed 30 min after each feeding. To calculate feed intake, it was assumed that the feed to water ratio in feed refusals was 1:2. The data of pigs refusing 15% or more of their daily feed allowance during a balance period were excluded from the results obtained in that balance period. In the remaining pigs, feed refusals were very low. The measured feed intake of the remaining pigs on the R regime was 96.0% of that of pigs receiving the A regime. All pigs had free access to drinking water.

**Table 1.** Composition of experimental diets (g/kg)

	Diet for the A regime	Diet for the R regime
Ingredient composition, g/kg		
Basal mixture^1^		
Wheat starch	247.0	261.6
Pregelatinized potato starch	236.5	250.7
Oat hulls	100.0	105.9
Dextrose	100.0	105.9
Beet pulp	50.0	52.9
Soybean oil	31.2	33.1
Potassium carbonate	10.2	10.8
Monocalcium phosphate	11.9	12.6
Limestone	14.3	15.1
Sodium chloride	3.9	4.1
Vitamin and mineral premix^2^	5.0	5.3
Titanium dioxide	4.0	4.0
Protein-containing ingredients		
Casein	52.0	38.6
Wheat gluten meal	94.9	70.4
Potato protein^3^	37.2	27.6
l-Lys HCl	1.7	1.3
l-Thr	0.2	0.1
Analyzed composition, g/kg		
DM	901	901
Crude ash	50.1	52.2
Crude protein (N × 6.25)	165.8	125.4
Crude fat	24.5	24.8
Starch	420.4	438.1
Sugar	93.8	103.8
Titanium	2.59	2.71
GE, MJ/kg	16.71	16.53
NE, MJ/kg^4^	10.58	10.62
AID lysine^4^	8.5	6.4
AID methionine + cystine^4^	5.4	4.1
AID threonine^4^	5.6	4.2
AID tryptophan^4^	1.6	1.2
AID isoleucine^4^	6.7	5.0

^1^Two levels of dietary protein supply, A or R (70% of A), were used in the study, at a similar daily supply of other nutrients. In the R supply, the proportion of protein-containing ingredients in the diet was reduced by 30% relative to the proportion in the A diet. In order to supply all pigs, relative to their metabolic BW, with the same amount of basal ingredients and nutrients, the feed allowance of pigs on the R regime was 94.4% of that of the A regime.

^2^Provided per kilogram of adequate protein diet: vitamin A, 7,000 IU; vitamin D3, 1,700 IU; vitamin E, 100 IU; vitamin K_3_, 1.5 mg; thiamine, 0.75 mg; riboflavin, 5.0 mg; d-pantothenic acid, 11 mg; niacin, 60 mg; vitamin B_12_, 18 μg; folic acid, 2.5 mg; pyridoxine, 1.0 mg; choline chloride, 100 mg; Fe (FeSO_4_–H_2_O), 75 mg; Cu (CuSO_4_–5H_2_O), 10 mg; Zn (ZnSO_4_–H_2_O), 65 mg; Mn (MnO), 30 mg; I (KI), 0.75 mg; and Se (Na_2_SeO_3_–5H_2_O), 0.3 mg.

^3^Protastar, Avebe Feed, Veendam, The Netherlands.

^4^NE and apparent ileal digestible (AID) lysine based on CVB (2016) feed table. Chemical composition and nutritional value of feedstuffs.

### Observations and chemical analysis

#### Performance

BW of the pigs was determined at birth, day 28 (weaning), day 63 (moving to the GF room), day 98 (start of the experiment), days 104 and 114 (start of both periods of adaptation to the experimental diets), and day 125 (end of the experimental period). Feed refusals were collected twice daily and feed intake per day was calculated per pig.

#### Nitrogen balance

Pigs were equipped with a Velcro support system to allow the separate collection of feces (Van Kleef et al., 1994) and urine. Feces and urine were collected quantitatively from each pig during two periods of five subsequent days each. Feces was collected daily, weighed, and stored at −20 °C pending analysis. Urine was collected via funnels, which were sprayed with an acetic acid buffer (sodium acetate 0.08 M, formic acid 0.025 M, and acetic acid 0.013 M), into buckets containing 35 mL of sulfuric acid (4.5 M), to maintain a pH < 3 to prevent volatilization of NH_3_. Urine was collected daily from the buckets, weighed, pooled per pig per N balance period, mixed, sampled, and stored at −20 °C pending analysis.

#### Chemical analysis

Representative feed samples were obtained by pooling small aliquots of feed collected from each bag used during the trial period. Fecal samples were pooled per animal per balance period, homogenized, sampled, freeze-dried, and ground to pass a 1-mm mesh sieve using a Retsch ZM 100 mill (Retsch GmbH, Haan, Germany) before analysis. Feed samples were analyzed for dry matter (**DM**), ash, N, crude fat, starch, sugar, energy, and titanium. Fecal samples were analyzed for DM, N, energy, and titanium. Dry matter was analyzed by drying at 103 °C (ISO 6496), ash by combustion to a constant weight at 550 °C (ISO, 5984), N by using the Dumas method (ISO 16634-1), crude fat after hydrolysis (ISO, 6492), and energy by using an adiabatic bomb calorimeter (ISO 9831). Starch in feed samples was determined spectrophotometrically (Evolution 201; Thermo Scientific, Waltham, MA, USA) after enzymatic conversion into glucose (ISO 15914). Determination of sugars was based on the method described by Van Vuuren et al. (1993). Titanium was also determined spectrophotometrically (Evolution 201; Thermo Scientific, Waltham, MA, USA) after hydrolysis with H_2_SO_4_ (Tecator digestion system; FOSS, Hillerød, Denmark) and subsequent addition of peroxide (Myers et al., 2004). Apparent total tract digestibility (**ATTD**) for DM, N, and energy was calculated using TiO_2_ as an indigestible marker with the following equation: ATTD (%) = 100% - [titanium_diet_/ titanium_freeze -dried feces_] × [DM, N, energy_freeze -dried feces_/ DM, N, energy_diet_] × 100% (Stein et al., 2007).

Urine samples were analyzed for total N, urea, and creatinine. Total N was analyzed by using the Dumas method (ISO 16634-1). Urea was analyzed enzymatically by the urease method (Tabacco et al., 1979), and creatinine was analyzed enzymatically by the Jaffé method (Cook, 1971).

#### Blood samples

At the end of both balance periods, blood samples were collected from the jugular vein. The interval between feeding time and time of blood sampling was 1.5 to 3 h. Per sampling moment, two 9-mL plasma tubes (Vacuette, Greiner Bio-One, Kremsmünster, Austria) per pig were filled and allowed to clot for 1 h at room temperature. Plasma was collected after centrifugation for 15 min at 2,000 × *g* and was stored at −80 °C pending analyses on free AA, insulin, glucose, urea, insulin-like growth factor (**IGF**-1), creatinine, and nontargeted metabolomics. Free AA were analyzed according to ISO 3903. Insulin was analyzed with the porcine insulin radioimmunoassay kit (cat no PI-12K; EMD Millipore Corporation, USA). Glucose was determined spectrophotometrically (Olympus AU680; Beckman Coulter, UK) using the hexokinase method. Urea was determined with the urease–glutamate dehydrogenase method (Olympus AU680; Beckman Coulter, UK). IGF-1 was analyzed using the chemiluminescence method (IMMULITE 2000, Siemens, Germany). Creatinine was analyzed using the ADVIA 1650 Chemistry system (Siemens Diagnostics, Tarrytown, NY, USA) according to the manufacturer’s instructions (Siemens Diagnostics Clinical Methods for ADVIA 1650).

#### Metabolomics

In both balance periods, at day 4 between 0900 and 1000 hours, three representative 1.5 mL urine samples per pig were taken from the buckets with urine collected from 0800 hours that morning. The samples were stored at −80 °C pending analyses. Nontargeted metabolomics analyses were performed in the urine and plasma samples. The preparation of the urine and plasma samples before data processing and metabolite identification is described in Van der Peet-Schwering et al. (2020). As quality controls (**QC**s), a blank sample (5% acetonitrile) was injected after each four samples to evaluate potential cross-contamination from samples and four pooled urine and plasma samples were injected four times during the chromatographic run to evaluate the analytical system performance, loss of sensitivity, and system reproducibility during the run. The blank samples showed no addition of peaks indicating that no cross-contamination between samples occurred. Furthermore, the chromatograms of the reinjected QC samples were indistinguishable and they showed close clustering in principal component analysis (**PCA**) scores plots verifying the stability and reproducibility of the analytical system.

### Data processing and metabolite identification

Mass spectra were calibrated and converted into the mzXML file format. Mass features of the urine and plasma samples were extracted using the R-based XCMS package (Smith et al., 2006) where peak picking was performed using the “centWave” method and retention time (**RT**) was aligned using “Obiwarp.” Missing values were substituted using the “fillpeaks” method and isotopes were annotated using CAMERA (Kuhl et al., 2012). Exported data were filtered to eliminate peaks present in blanks, and RT was truncated to contain only portions with chromatographic peaks and masses higher than 700 m/z were discarded. The datasets were normalized using the van der Kloet procedure (van der Kloet et al., 2009) based on the QC samples.

Initial PCA was performed to check the quality of the data sets and eliminate potential outliers. Partial least-squares discriminant analysis (**PLS-DA**) models were built to determine the metabolites responsible for the differences between pigs with HBV and LBV, with EBV as a continuous variable, and for the differences between pigs on the A and R feeding regime. Validation of the models was performed using full cross-validation (leave-one-out). Outliers were detected based on the residual variance and the Hotelling’s T^2^ plot. Models were assessed using the explained variation in Y, plots depicted actual and predicted values, and the proportion of variation explained (*R*^2^). Variable selection was done by excluding low-importance variables based on the variable importance in projection (**VIP**) scores. Variables for identification were selected using VIP scores and scaled regression coefficients.

Compounds in both urine and plasma samples were identified based on queries in the METLIN (http://metlin.scripps.edu/), Human Metabolome Database (http://www.hmdb.ca/), and LIPID MAPS (http://www.lipidmaps.org/) online databases for obtaining possible chemical structures using accurate mass and mass spectrometric fragmentation patterns. The identification of annotated compounds was confirmed with standards, when available, on the same analytical system under the same conditions.

### Statistical analyses

The experimental data were analyzed by analysis of variance (ANOVA) with balance period as a random factor; pig as an experimental unit; and BiW, EBV, dietary protein regime, sequence of offering the experimental diets, and the interaction between BiW and EBV, BiW and dietary protein regime, and EBV and dietary protein regime as explanatory factors using GenStat statistical software (GenStat 19th edition). The same statistical model, using the lme4 package in R (Bates et al., 2015), was used to analyze the metabolite ion intensities in urine and plasma samples that were found discriminating between EBV and dietary protein regime via multivariate analyses. The R package “car” (Fox and Weisberg, 2011) was used for estimating the *P*-values. Least squares means were computed using the R package “lsmeans” (Lenth, 2016). Differences were considered significant at *P* < 0.05, whereas 0.05 ≤ *P* ≤ 0.10 was considered as a tendency. Fisher’s protected least significant difference was used to identify pairwise differences (*P* < 0.05) between LBW and HBW pigs, LBV and HBV pigs, and dietary treatments. The sequence of offering the experimental diets did not affect any of the measured parameters.

## Results

One HBW–LBV pig died during the N balance study. In the first balance period data of two LBW–HBV pigs and one HBW–HBV pig and, in the second balance period data of two LBW–LBV pigs were excluded from the results because these pigs refused more than 15% of their daily feed allowance. This means that during balance period 1, the data of 18 LBW (9 on the A regime and 9 on the R regime), 18 HBW (8 on the A regime and 10 on the R regime), 19 LBV (9 on the A regime and 10 on the R regime), and 17 HBV (8 on the A regime and 9 on the R regime) pigs were included in the results. During balance period 2, the data of 18 LBW (9 on the A regime and 9 on the R regime), 19 HBW (10 on the A regime and 9 on the R regime), 17 LBV (9 on the A regime and 8 on the R regime), and 20 HBV (10 on the A regime and 10 on the R regime) pigs were included in the results.

At all weighing moments, the HBW pigs were significantly (*P* < 0.001) heavier than the LBW pigs, whereas there were no differences in BiW and BW between the HBV and LBV pigs (Table 2). The difference in BiW between the LBW and HBW pigs was 0.76 kg (1.04 vs. 1.80 kg). On day 98 (start of the experiment) and day 125 (end of the experiment), the differences in BW between the LBW and HBW pigs were 10.9 and 13.5 kg, respectively.

**Table 2.** BW development^1^ (kg) of male growing pigs with an LBW or HBW and an LBV or HBV for protein deposition

	BiW			EBV			*P*-value		
	LBW	HBW	SEM	LBV	HBV	SEM	BiW	EBV	BiW × EBV
Birth	1.04	1.80	0.03	1.41	1.43	0.03	<0.001	0.53	0.58
Day 28	6.58	8.79	0.24	7.59	7.72	0.24	<0.001	0.71	0.95
Day 63	19.4	26.1	0.48	22.6	22.8	0.48	<0.001	0.77	0.47
Day 98	49.0	59.9	0.92	54.5	54.6	0.92	<0.001	0.81	0.41
Day 104	53.2	62.7	1.09	58.0	57.9	1.09	<0.001	0.97	0.33
Day 114	60.6	70.5	1.33	65.8	65.3	1.33	<0.001	0.80	0.38
Day 125	69.8	83.3	1.30	77.0	76.1	1.30	<0.001	0.62	0.21

^1^BW of the pigs was determined at birth, day 28 (weaning), day 63 (moving to the GF room), day 98 (start N balance study), days 104 and 114 (start of both periods of adaptation to the experimental diets), and day 125 (end of the experimental period).

Dietary N intake, fecal N excretion, and urinary N excretion (in g/[kg BW^0.75^ · d]; Table 3) were not affected by the BiW of the pigs. Moreover, N retention (in g/[kg BW^0.75^ · d]), N efficiency based on total N intake (= 100% × N retention / N intake), and N efficiency based on digestible N intake (= 100% × N retention/digestible N intake) were not affected by BiW of the pigs. Dietary N intake and fecal N excretion were not affected by EBV. Urinary N excretion was similar in LBV and HBV pigs on the R regime but lower in HBV than LBV pigs on the A regime. Nitrogen retention and N efficiency based on total N intake and on digestible N intake were similar in LBV and HBV pigs on the R regime but higher in HBV than LBV pigs on the A regime. Restricting dietary protein supply reduced the dietary N intake (*P* < 0.001), the urinary N excretion (*P* < 0.001), and the N retention (*P* < 0.001). Moreover, it increased the N efficiency based on total N intake and on digestible N intake in LBV pigs but not in HBV pigs.

**Table 3.** Effects of BiW, EBV for protein deposition, and dietary protein supply on nitrogen (N) balance parameters (g/[kg BW^0.75^ · d]) in male growing pigs

	BiW^1^			EBV^1^			Dietary protein supply^1^			*P*-value		
	LBW	HBW	SEM	LBV	HBV	SEM	A	R	SEM	BiW	EBV	Diet
N intake	1.906	1.909	0.009	1.908	1.907	0.009	2.201	1.614	0.008	0.78	0.91	<0.001
Fecal N	0.171	0.159	0.005	0.168	0.162	0.005	0.160	0.169	0.004	0.12	0.48	0.16
Urinary N^2^	0.694	0.731	0.019	0.743	0.682	0.019	0.882	0.543	0.014	0.19	0.03	<0.001
N retention^3^	1.041	1.020	0.022	0.998	1.063	0.022	1.159	0.902	0.016	0.51	0.04	<0.001
N efficiency^4,5^, %	54.8	53.7	1.03	52.7	55.8	1.03	52.7	55.8	0.67	0.42	0.04	0.003
N efficiency^6,7^	60.8	59.0	1.05	58.4	61.5	1.05	57.2	62.7	0.71	0.23	0.04	<0.001

^1^Number of observations: 36 and 37 in LBW and HBW pigs, respectively; 36 and 37 in LBV and HBV pigs, respectively; 36 and 37 on the A and R regime, respectively.

^2^An EBV × Diet interaction was observed (*P* = 0.006): A regime: urinary N in pigs with LBV and HBV is 0.942 and 0.822 g/(kg BW^0.75^ · D), respectively; R regime: urinary N in pigs with LBV and HBV is 0.544 and 0.542 g/(kg BW^0.75^ · d), respectively.

^3^An EBV × Diet interaction was observed (*P* = 0.05): A regime: N retention in pigs with LBV and HBV is 1.104 and 1.214 g/(kg BW^0.75^ · d), respectively; R regime: N retention in pigs with LBV and HBV is 0.892 and 0.912 g/(kg BW^0.75^ · d), respectively.

^4^N efficiency = 100% × N retention/N intake.

^5^An EBV × Diet interaction was observed (*P* = 0.04): A regime: N efficiency in pigs with LBV and HBV is 50.2% and 55.2%, respectively; R regime: N efficiency in pigs with LBV and HBV is 55.2% and 56.3%, respectively.

^6^N efficiency = 100% × N retention / fecal digestible N intake.

^7^An EBV × Diet interaction was observed (*P* = 0.02): A regime: N efficiency in pigs with LBV and HBV is 54.4% and 60.0%, respectively; R regime: N efficiency in pigs with LBV and HBV is 62.3% and 63.0%, respectively.

Digestibility of DM, N, and gross energy (**GE**) (Table 4) was not affected by BiW and EBV of the pigs. Restricting dietary protein supply reduced the digestibility of DM (*P* < 0.001), N (*P* < 0.001), and GE (*P* < 0.001). 

**Table 4.** Effects of BiW, EBV for protein deposition, and dietary protein supply on total tract digestibility of dry matter (DM), nitrogen (N), and gross energy (GE) (%) in male growing pigs

	BiW^1^			EBV^1^			Dietary protein supply^1^			*P*-value		
	LBW	HBW	SEM	LBV	HBV	SEM	A	R	SEM	BiW	EBV	Diet
DM	88.0	88.3	0.15	88.0	88.3	0.15	88.7	87.6	0.17	0.14	0.28	<0.001
N	90.8	91.5	0.29	91.0	91.3	0.29	92.7	89.5	0.22	0.12	0.43	<0.001
GE	88.2	88.8	0.23	88.4	88.5	0.23	89.1	87.9	0.19	0.07	0.77	<0.001

^1^Number of observations: 36 and 37 in LBW and HBW pigs, respectively; 36 and 37 in LBV and HBV pigs, respectively; 36 and 37 on the A and R regime, respectively.

Urinary concentration of N, urea, and creatinine (Table 5) was not affected by BiW and EBV of the pigs. Restricting dietary protein supply reduced the urinary concentration of N and urea (*P* < 0.001). Urinary concentration of creatinine was not affected by dietary protein supply.

**Table 5.** Effects of BiW, EBV for protein deposition, and dietary protein supply on the concentration of nitrogen, urea, and creatinine in the urine of male growing pigs

	BiW^1^			EBV^1^			Dietary protein supply^1^			*P*-value		
	LBW	HBW	SEM	LBV	HBV	SEM	A	R	SEM	BiW	EBV	Diet
Nitrogen, g/kg	4.24	3.61	0.28	3.91	3.93	0.28	4.78	3.07	0.15	0.11	0.96	<0.001
Urea, mmol/L	117.3	99.3	7.4	108.4	108.2	7.4	135.5	81.1	4.5	0.10	0.98	<0.001
Creatinine, mmol/L	7.05	6.39	0.45	6.27	7.17	0.45	6.65	6.79	0.25	0.31	0.17	0.70

^1^Number of observations: 36 and 37 in LBW and HBW pigs, respectively; 36 and 37 in LBV and HBV pigs, respectively; 36 and 37 on the A and R regime, respectively.

The concentration of insulin, urea, α amino N, and creatinine in blood plasma was not affected by the BiW of the pigs. The concentration of glucose in blood plasma was lower in HBW than in LBW pigs (*P* = 0.007; Table 6). The concentration of IGF-1 in blood plasma was similar in LBW and HBW pigs on the A regime but higher in HBW than LBW pigs on the R regime. EBV for protein deposition did not affect the concentration of insulin, glucose, urea, and α amino N in blood plasma. The concentration of creatinine in blood plasma was higher in HBV than LBV pigs (*P* = 0.002). The concentration of IGF-1 in blood plasma was similar in LBV and HBV pigs on the R regime but higher in HBV than LBV pigs on the A regime. Restricting dietary protein supply decreased the concentration of insulin (*P* = 0.06) and urea (*P* < 0.001) and increased the concentration of creatinine (*P* < 0.001) in blood plasma. Moreover, it decreased the IGF-1 concentration in blood plasma in LBW pigs and in HBV pigs but not in HBW pigs and in LBV pigs. The concentration of glucose and α amino N in blood plasma was not affected by dietary protein supply.

**Table 6.** Effects of BiW, EBV for protein deposition, and dietary protein supply on the concentration of insulin, glucose, urea, IGF-1, α amino nitrogen (N), and creatinine in the blood plasma of male growing pigs

	BiW^1^			EBV^1^			Dietary protein supply^1^			*P*-value		
	LBW	HBW	SEM	LBV	HBV	SEM	A	R	SEM	BiW	EBV	Diet
Insulin, uU/mL	28.6	31.8	2.26	31.0	29.4	2.26	32.5	27.9	1.68	0.32	0.63	0.06
Glucose, mmol/L	6.43	5.90	0.13	6.02	6.31	0.13	6.24	6.10	0.09	0.007	0.13	0.29
Urea, mmol/L	3.19	3.12	0.10	3.21	3.10	0.10	3.67	2.64	0.06	0.65	0.41	<0.001
IGF-1^2,3^, µg/L	184	195	10.6	182	197	10.6	200	179	3.7	0.46	0.32	<0.001
α amino N, mmol/L	7.37	7.37	0.14	7.45	7.29	0.14	7.36	7.38	0.11	1.00	0.42	0.88
Creatinine, µmol/L	95.7	96.2	2.4	90.3	101.5	2.4	91.0	100.8	1.7	0.88	0.002	<0.001

^1^Number of observations: 36 and 37 in LBW and HBW pigs, respectively; 36 and 37 in LBV and HBV pigs, respectively; 36 and 37 on the A and R regime, respectively.

^2^A BiW × Diet interaction was observed (*P* = 0.02): A regime: IGF-1 in pigs with LBW and HBW is 200 and 199 µg/L, respectively; R regime: IGF-1 in pigs with LBW and HBW is 167 and 191 µg/L, respectively.

^3^An EBV × Diet interaction was observed (*P* = 0.02): A regime: IGF-1 in pigs with LBV and HBV is 186 and 214 µg/L, respectively; R regime: IGF-1 in pigs with LBV and HBV is 178 and 180 µg/L, respectively.

**Figure 1.**
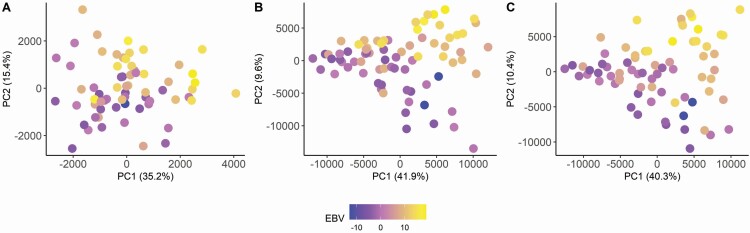
PC plot of plasma samples in positive mode (A), urine samples in positive mode (B), and urine samples in negative mode (C). Colors indicate the different EBV for protein deposition of the pigs.

After preprocessing of the metabolomics data, eight models were built to identify metabolites in plasma and urine discriminating the experimental factors EBV of the pigs and dietary protein supply. Attempts to build a model discriminating pigs based on BiW were not successful. The composed models were evaluated based on several criteria (Table 7). Valid models were obtained in most of the cases, except for the plasma metabolome obtained in negative mode in relation to EBV. Figure 1 shows the PLS-DA scores plots of the models on EBV for the plasma samples in the positive mode and the urine samples in the positive and negative modes. For plasma, the combined explained variation for principal component (**PC**) 1 and PC2 was 50.6%. The model stability, however, was low (*R*^2^ = 0.31). The 10 metabolites within each ionization mode with the highest VIP scores and metabolites differing significantly between HBV and LBV pigs are presented in Table 8. Other metabolites contributing to the discrimination but not differing significantly between HBV and LBV pigs are presented in Supplementary Table S1. The level of a hexose, presumably glucose, in plasma was significantly higher in HBV than in LBV pigs. The same was observed for the free AA methionine and phenylalanine, whereas leucine also contributed to the separation without differing significantly between HBV and LBV pigs. Furthermore, four lysophosphatidylcholines (**LPC**) and creatinine contributed to the discrimination of HBV and LBV pigs. The stability of the models discriminating urine samples was higher, *R*^2^ = 0.56 and *R*^2^ = 0.58, and the combined explained variation for PC1 and PC2 was 50.7% and 51.5% for the data obtained in the negative and positive mode, respectively. In urine, creatinine, 7-methylguanine, three unidentified metabolites, and five metabolites conjugated to glucuronic acid differed significantly between HBV and LBV pigs. The microbial metabolite *p*-cresol conjugated with either glucuronic acid or sulfate tended (*P* < 0.10) to differ between HBV and LBV pigs. Other metabolites of microbial origin, hippuric acid, phenylacetylglycine, and cinnamoylglycine, also contributed to the discrimination of pigs according to EBV class but the concentration of the metabolites did not differ significantly between EBV class.

**Table 7.** Models used in PLS-DA of the metabolome profiles in plasma and urine

Model	No. of observations	No. of observations after outlier removal	Initial feature no.	Final feature no.	PC no.	evY^1^calibrated	evY validated	*R* ^2^
EBV								
Urine negative	78	72	2,374	414	5	88.4	56.9	0.557
Urine positive	78	75	4,302	823	5	86.8	59.1	0.578
Plasma negative	78		194		1	33.7	−28.0	−0.022
Plasma positive	67	65	482	63	4	60.0	29.8	0.311
Dietary protein supply								
Urine negative	78	72	2,374	426	3	82.4	64.9	0.639
Urine positive	78	73	4,302	842	3	82.8	65.4	0.646
Plasma negative	78	78	194	33	3	64.4	51.2	0.502
Plasma positive	67	65	482	61	4	76.5	56.0	0.558

^1^Explained variation in Y.

**Table 8.** List of plasma and urine metabolites identified from metabolomics analysis discriminating between male growing pigs differing in EBV for protein deposition^1^

Metabolite	Adduct	Mode^2^	RT	M to Z ratio	VIP	Level of ID^3^	Fold change^4^		*P*-value^5^		
							D	EBV	D	EBV	D × EBV
Plasma											
LPC 16:0	[M + H]+	Pos	9.64	496.341	6.57	3	−1.07	1.02	0.50	0.47	0.80
Leucine	[Fragment]	Pos	1.23	86.097	5.65	1	−1.12	−1.01	0.01	0.56	0.27
Phenylalanine	[Fragment]	Pos	2.11	120.081	5.03	1	−1.09	−1.04	0.05	0.02	0.25
LPC 18:2	[M + H]+	Pos	9.21	520.341	3.92	2	1.04	1.06	0.89	0.10	0.83
Phenylalanine	[M + H]+	Pos	2.11	166.087	3.46	1	−1.09	−1.04	0.05	0.01	0.24
LPC 18:0	[M + H]+	Pos	10.96	524.372	3.41	3	−1.02	1.08	0.73	0.30	0.45
Leucine	[M + H]+	Pos	1.25	132.102	3.11	1	−1.12	−1.01	0.004	0.52	0.19
LPC 18:1	[M + H]+	Pos	9.98	522.357	2.65	2	1.01	1.08	0.76	0.23	0.45
Creatinine	[M + H]+	Pos	0.70	114.066	2.60	1	1.09	−1.11	0.002	0.14	0.46
C_6_H_12_O_6_	[M + Na]+	Pos	0.70	203.053	2.54	3	−1.00	−1.05	0.83	0.04	0.85
C_6_H_12_O_6_	[2M + Na]+	Pos	0.70	383.117	1.01	3	1.00	−1.07	0.83	0.02	0.67
Methionine	[M+ H ]+	Pos	0.94	150.059	0.41	1	−1.09	−1.07	0.03	0.01	0.07
Phenylalanine	[Fragment]	Pos	2.11	103.055	0.32	1	−1.10	−1.04	0.06	0.02	0.29
Urine											
*p*-Cresol glucuronide	[M − H]−	Neg	4.01	283.082	13.57	2	1.06	−1.08	0.38	0.08	0.80
2-Methylbutyrylglycine/valerylglycine	[M − H]−	Neg	3.00	158.082	12.42	2	−1.11	1.17	0.90	0.34	0.15
Phenylacetylglycine	[M − H]−	Neg	3.85	192.066	11.64	1	1.04	−1.08	0.46	0.25	0.62
Citric acid	[M − H]−	Neg	0.94	191.020	11.60	1	−1.17	1.02	0.60	0.86	0.79
Hippuric acid	[M − H]−	Neg	3.51	178.051	9.90	1	1.02	−1.07	0.43	0.43	0.39
*p*-Cresol sulfate	[M − H]−	Neg	4.04	187.007	8.26	1	1.04	−1.09	0.79	0.09	0.60
Sulfated steroid	[M − H]−	Neg	6.24	367.158	8.21	3	1.04	−1.22	0.76	0.57	0.43
Sulfated compound	[M − H]−	Neg	3.57	167.038	7.02	3	1.11	1.08	0.63	0.96	0.93
Glucuronide conjugate	[M − H]−	Neg	5.81	415.197	6.43	3	1.08	−1.03	0.80	0.12	0.46
Glucuronide conjugate	[M − H]−	Neg	4.26	449.202	6.42	3	1.06	−1.16	0.33	0.11	0.93
Glucuronide conjugate	[M − H]−	Neg	5.26	433.208	5.49	3	1.00	−1.32	0.99	0.001	0.76
Glucuronide conjugate	[M − H]−	Neg	4.97	431.192	5.05	3	1.02	−1.25	0.85	0.01	0.34
Glucuronide conjugate	[M − H]−	Neg	6.12	461.239	3.00	3	1.11	−1.50	0.97	0.0002	0.82
Glucuronide conjugate	[M − H]−	Neg	6.26	417.213	2.32	3	1.16	−1.21	0.49	0.01	0.59
Phenylacetylglycine	[M + H]+	Pos	3.86	194.081	40.04	1	−1.01	−1.05	0.26	0.39	0.18
2-Methylbutyrylglycine/valerylglycine	[M + H]+	Pos	3.00	160.097	15.23	2	−1.11	1.18	0.76	0.47	0.12
Hippuric acid	[Fragment]	Pos	3.50	105.034	14.92	1	1.04	−1.07	0.30	0.48	0.31
Hippuric acid	[M + H]+	Pos	3.50	180.066	14.70	1	1.05	−1.06	0.28	0.53	0.27
Creatinine	[M + H]+	Pos	0.72	114.066	12.50	1	1.00	−1.13	0.85	0.12	0.34
Cinnamoylglycine	[Fragment]	Pos	4.77	131.049	9.26	1	1.16	1.53	0.63	0.80	0.89
Unidentified	[M + H]+	Pos	4.96	510.272	9.25	4	1.063	−1.12	0.97	0.65	0.38
Unidentified	[M + H]+	Pos	5.37	130.065	9.24	4	−1.13	1.00	0.72	0.34	0.81
Piperidine	[M + H]+	Pos	0.83	86.097	8.05	1	1.14	−1.44	0.08	0.22	0.25
l-Formylkynurenine	[Fragment]	Pos	3.93	144.045	7.38	2	−1.02	1.07	0.87	0.50	0.64
Creatinine	[M + Na]+	Pos	0.70	136.048	5.36	1	1.11	−1.30	0.94	0.02	0.09
Unidentified	[M + H]+	Pos	5.34	444.312	3.81	4	−1.18	−1.40	0.25	0.01	0.91
Unidentified	[M + H]+	Pos	5.95	635.379	3.58	4	−1.48	−1.87	0.01	0.001	0.40
Creatinine	[2M + Na]+	Pos	0.70	249.107	3.50	1	1.18	−1.42	0.70	0.01	0.15
Melatonin glucuronide	[M + H]+	Pos	3.82	409.160	3.23	2	−1.01	−1.45	0.99	0.01	0.84
7-Methylguanine	[M + H]+	Pos	0.90	166.072	2.76	2	1.10	−1.16	0.45	0.02	1.00
Unidentified	[M + H]+	Pos	5.95	307.202	2.52	4	−1.42	−1.63	0.02	0.001	0.47

^1^The table encompasses the 10 metabolites with the highest VIP scores in each sample type and ionization mode and metabolites differing significantly between HBV and LBV.

^2^Pos, positive; Neg, negative.

^3^Level of identification: (**ID**): identified metabolites (level 1), putatively annotated compounds (level 2), putatively characterized compound classes (level 3), and unknown compounds (level 4).

^4^Fold change in metabolite intensity: dietary protein supply (D: A [reference] vs. R regime) and EBV (high [reference] vs. low).

^5^ *P*-values for the main effects of dietary protein supply (D: A or R regime [70% of A]), EBV, and the interactive effect of dietary protein supply and EBV (D × EBV).

The PLS-DA scores plots of the plasma and urinary metabolome of pigs fed according to the A or R regime are shown in Supplementary Figure S1. The stability of the models on the dietary treatment was higher than for the models on the effect of EBV (Table 7). The variation explained by PC1 and PC2 was, however, within the same range (34% to 55%). The 10 metabolites within each ionization mode with the highest VIP scores and metabolites differing significantly between pigs fed on the A or R regime are presented in Table 9. In plasma, AA and the derivatives thereof were the main metabolites differing significantly between pigs fed according to the A or R regime, with concentrations all being higher in the animals fed the A regime. Other metabolites differing significantly, i.e., azelaic acid, creatinine, and 4-trimethylammoniobutanoic acid, were all higher in animals fed the R regime. The urinary metabolites contributing to the discrimination in the PLS-DA model to discriminate according to feeding regime were predominantly of microbial origin (*p*-cresol glucuronide, *p*-cresol sulfate, hippuric acid, and indoxylsulfuric acid), values as such, however, not being significantly different between the A and R regime. Taurine, acetyl-dl-l-leucine, picolinoylglycine, a sulfated compound, and two unidentified compounds differed significantly between the A and R regime, and values were all higher in animals fed the A regime, whereas oxindole, a microbial metabolite, was higher in animals fed the R regime.

**Table 9.** List of plasma and urine metabolites identified from metabolomics analysis discriminating between male growing pigs that were fed according to A or R regime^1^

Metabolite	Adduct	Mode^2^	RT	M to Z ratio	VIP	Level of ID^3^	Fold change^4^		*P*-value^5^		
							D	EBV	D	EBV	D × EBV
Plasma											
Tyrosine^6^	[M − H]−	Neg	1.16	180.067	5.32	1	−1.19	1.01	0.001	0.43	0.24
Lactic acid	[M − H]−	Neg	0.97	89.024	4.08	1	1.03	1.02	0.98	0.43	0.37
Indoxylsulfuric acid	[M − H]−	Neg	3.54	212.002	3.71	1	−1.63	1.23	0.12	0.21	0.91
Tryptophan	[M − H]−	Neg	2.88	203.083	3.40	1	−1.17	1.04	0.01	0.68	0.77
Phenylalanine^6^	[M − H]−	Neg	2.11	164.072	1.78	1	−1.16	−1.02	0.002	0.05	0.61
Leucine	[M − H]−	Neg	1.27	130.087	1.73	1	−1.18	1.01	0.0001	0.31	0.32
Ketoisoleucine	[M − H]−	Neg	3.21	129.056	1.54	1	−1.13	−1.08	0.002	0.53	0.68
Taurodeoxycholic acid	[M − H]−	Neg	5.87	498.290	1.22	2	−2.20	1.09	0.004	0.47	0.92
Ketoleucine	[M − H]−	Neg	3.54	129.056	1.21	1	−1.03	1.06	0.09	0.54	0.22
Unidentified	[M − H]−	Neg	0.71	215.033	0.98	4	−1.05	−1.04	0.11	0.11	0.93
Azelaic acid	[M − H]−	Neg	4.90	187.098	0.60	1	1.12	−1.02	0.001	0.43	0.51
2-Hydroxybutyric acid	[M − H]−	Neg	1.56	103.040	0.54	1	−1.41	−1.07	0.01	0.76	0.77
Hydroxyphenyllactic acid^6^	[M − H]−	Neg	2.92	181.051	0.35	1	−1.21	−1.13	0.002	0.16	0.77
Tyrosine^6^	[2M + Na]−	Neg	1.15	383.122	0.34	1	−1.20	1.03	0.01	0.55	0.31
Unidentified	[M − H]−	Neg	1.17	189.041	0.25	4	1.14	−1.11	0.001	0.87	0.37
Indolelactic acid	[M − H]−	Neg	4.61	204.067	0.19	2	−1.19	−1.07	0.03	0.88	0.68
4-Trimethylammonio-butanoic acid	[Fragment]	Pos	2.57	100.112	5.79	2	1.30	1.01	0.001	0.98	0.06
Leucine	[Fragment]	Pos	1.23	86.097	5.10	1	−1.12	−1.00	0.005	0.57	0.54
LPC 18:2	[M + H]+	Pos	9.21	520.341	3.27	2	1.01	1.03	0.88	0.32	0.74
Phenylalanine	[Fragment]	Pos	2.11	120.081	2.68	1	−1.10	−1.05	0.05	0.01	0.49
Leucine	[M + H]+	Pos	1.25	132.102	2.64	1	−1.12	−1.00	0.004	0.53	0.38
4-Trimethylammonio-butanoic acid	[M + H]+	Pos	2.57	146.118	2.59	2	1.31	1.02	0.003	0.94	0.04
Unidentified	[M + H]+	Pos	4.58	239.090	2.53	4	−1.00	−1.00	0.31	0.82	0.14
Creatine	[M + H]+	Pos	0.71	132.077	2.22	1	−1.12	1.09	0.78	0.55	0.86
LPC 16:0	[M + H]+	Pos	9.64	496.341	2.21	3	−1.10	−1.00	0.42	0.76	0.98
Tryptophan	[Fragment]	Pos	2.87	188.071	2.17	1	−1.17	1.01	0.16	0.65	0.67
Valine^6^	[M + Na]+	Pos	0.82	72.081	1.98	1	−1.17	−1.01	0.001	0.86	0.62
Proline	[M + H]+	Pos	0.73	116.071	1.54	1	−1.16	1.01	0.05	0.57	0.88
Tyrosine	[M + H]+	Pos	1.14	182.082	1.19	1	−1.14	−1.03	0.03	0.45	0.28
Creatinine	[M + H]+	Pos	0.70	114.066	0.97	1	1.09	−1.11	0.002	0.15	0.58
Urea^6^	[M + H]+	Pos	0.71	61.040	0.95	1	−1.21	1.04	0.05	0.47	0.86
Tyrosine	[Fragment]	Pos	1.14	165.055	0.95	1	−1.14	−1.03	0.03	0.47	0.29
Urine											
Citric acid^6^	[M − H]−	Neg	0.94	191.020	24.45	1	−1.11	1.04	0.86	0.98	0.73
*p*-Cresol glucuronide^6^	[M − H]−	Neg	4.01	283.082	16.86	2	1.06	−1.08	0.38	0.08	0.80
Phenylacetylglycine^6^	[M − H]−	Neg	3.85	192.066	15.32	1	1.04	−1.08	0.46	0.25	0.62
Hippuric acid^6^	[M − H]−	Neg	3.51	178.051	13.50	1	1.02	−1.07	0.43	0.43	0.39
2-Methylbutyrylglycine/valerylglycine^6^	[M − H]−	Neg	3.00	158.082	13.47	2	−1.11	1.17	0.90	0.34	0.15
Indoxylsulfuric acid	[M − H]−	Neg	3.46	212.002	11.59	1	1.04	−1.13	0.66	0.37	0.85
Hydroxyphenyllactic acid^1^	[M − H]−	Neg	2.89	181.051	8.13	1	−1.20	−1.12	0.09	0.03	0.75
Citric acid^6^	[Fragment]	Neg	0.94	111.009	7.67	1	−1.17	1.02	0.60	0.86	0.79
*p*-Cresol sulfate	[M − H]−	Neg	4.04	187.007	7.10	1	1.04	−1.09	0.79	0.09	0.60
*p*-Cresol glucuronide^6^	[2M − H]−	Neg	4.00	567.171	6.72	2	1.10	−1.12	0.46	0.08	0.89
Taurine	[M − H]−	Neg	0.68	124.007	4.53	1	−2.36	1.33	0.0002	0.01	0.01
Sulfated compound	[M − H]−	Neg	5.61	303.127	4.41	3	−1.35	−1.32	0.02	0.01	0.53
Acetyl-dl-Leucine^6^	[M − H]−	Neg	3.75	172.098	2.87	1	−1.31	1.01	0.03	0.85	0.98
Unidentified	[M − H]−	Neg	5.28	309.134	2.41	4	−1.62	−1.23	0.01	0.09	0.56
Phenylacetylglycine^6^	[M + H]+	Pos	3.86	194.081	22.54	1	1.02	−1.11	0.44	0.24	0.49
Hippuric acid^6^	[Fragment]	Pos	3.50	105.034	13.75	1	1.02	−1.08	0.40	0.41	0.46
Glucoronidated compound	[M + H]+	Pos	4.00	302.124	12.68	3	−1.32	1.08	0.36	0.68	0.22
Creatinine^6^	[M + H]+	Pos	0.72	114.066	12.58	1	1.02	−1.17	0.88	0.06	0.69
Hippuric acid^6^	[M + H]+	Pos	3.50	180.066	12.28	1	1.02	−1.07	0.38	0.45	0.43
7-Methylguanine	[M + H]+	Pos	0.90	166.072	6.62	2	1.12	−1.22	0.75	0.01	0.42
Picolinoylglycine	[M + H]+	Pos	2.98	181.061	6.09	2	−1.57	−1.33	0.02	0.05	0.86
2-Methylbutyrylglycine/valerylglycine^6^	[M + H]+	Pos	3.00	160.097	5.62	2	−1.14	1.20	0.81	0.49	0.17
Unidentified	[M + H]+	Pos	5.95	307.202	5.55	4	−1.42	−1.67	0.02	0.001	0.39
Creatinine^6^	[M + K]+	Pos	0.71	152.022	5.44	1	1.15	−1.27	0.08	0.04	0.95
Oxindole	[M + H]+	Pos	3.43	134.060	4.21	2	1.19	−1.17	0.03	0.69	0.49
Unidentified	[M + H]+	Pos	3.17	288.181	3.90	4	−1.34	−1.05	0.01	0.01	0.49

^1^The table encompasses the 10 metabolites with the highest VIP scores in each sample type and ionization mode and metabolites differing significantly between male growing pigs that were fed according to the A or R regime.

^2^Pos, positive; Neg, negative.

^3^Level of identification: identified metabolites (level 1), putatively annotated compounds (level 2), putatively characterized compound classes (level 3), and unknown compounds (level 4).

^4^Fold change in metabolite intensity: dietary protein supply (D: A [reference] vs. R regime) and EBV (high [reference] vs. low).

^5^ *P*-values for the main effects of dietary protein supply (D: A or R regime [R, 70% of A]), EBV, and the interactive effect of dietary protein supply and EBV (D × EBV).

^6^Metabolites also identified in our previous experiment (Van der Peet-Schwering et al. (2020).

## Discussion

The objective of this study was to determine the effects of BiW, EBV, and dietary protein supply on between animal variation in N retention, N efficiency, and concentrations of plasma and urinary metabolites related to N metabolism in growing pigs. The results of this study can contribute to identifying genotypic and phenotypic factors explaining variation in N efficiency in pigs in the growing phase and related biomarkers in blood and urine. Such factors can be used for the further development of precision feeding and new animal breeding concepts.

### Birth weight

BW and average daily gain (**ADG**) of the LBW pigs were lower than that of the HBW pigs of the same age. In several studies, it was shown that LBW pigs eat and grow less during the weaning and growing period (Rehfeldt and Kuhn, 2006; Bérard et al., 2010; Alvarenga et al., 2013; Douglas et al., 2013; Van der Peet-Schwering et al., 2013) and have a lower number of muscle fibers at birth (Gondret et al., 2005; Rehfeldt and Kuhn, 2006; Alvarenga et al., 2013) than HBW pigs. The lower number of muscle fibers resulting in lower muscle accretion and the lower feed intake in LBW pigs might result in a lower N retention compared with HBW pigs. In our study, inherent to the study design, the N retention (in g/d) indeed was lower in LBW than in HBW pigs (22.8 vs. 25.5 g/d; *P* < 0.001; Supplementary Table S2) due to a lower N intake (41.7 vs. 47.6 g/d; *P* < 0.001; Supplementary Table S2) of the LBW pigs compared with the HBW pigs. The N retention (in g/(kg BW^0.75^ · d), however, was not affected by BiW. Moreover, N efficiency (%) of pigs in the growing phase was not affected by BiW. Similar results were reported by Van der Peet-Schwering et al. (2020), who suggested that both the LBW and HBW growing pigs, fed restrictedly, did not reach the plateau of postnatal lean growth (maximum body protein deposition), resulting in the absence of a difference in N retention (in g/[kg BW^0.75^ · d]) and N efficiency (%).

Total tract digestibility of DM and N did not differ between LBW and HBW growing pigs, whereas total tract digestibility of GE tended to be higher in HBW pigs. Also, Van der Peet-Schwering et al. (2020) did not observe an effect of BiW on total tract digestibility of DM, N, and GE in growing pigs. D′Inca et al. (2011) demonstrated that intrauterine growth retardation in LWB pigs affected intestinal development, delaying the feeding-induced intestinal adaptation. The relative immaturity of the small intestine tissue may reduce digestive capacities during the suckling period, but the long-term effects are unknown (Morise et al., 2008). Considering our results, it seems that the digestive capacity of pigs of 14- to 18-wk-old is not affected by BiW.

The concentration of glucose in blood plasma was lower in HBW pigs than in LBW pigs, whereas the concentration of insulin in blood plasma did not differ between the HBW and LBW pigs. These results are in contrast with those of Van der Peet-Schwering et al. (2020), who observed a difference in plasma insulin concentration but not in plasma glucose concentration between HBW and LBW pigs in the fed state. Poore and Fowden (2004a, 2004b) found no effect of BiW on fasting glucose and insulin concentrations in pigs of 3 mo of age. Generally, plasma glucose and insulin concentrations rapidly increase after feeding and then gradually decrease. Differences between studies in insulin and glucose response after a meal may be related to differences in the interval between ingestion of a meal and blood sampling and in composition and size of the meal and may interfere with observed effects of experimental treatments.

The concentration of IGF-1 in blood plasma was higher in HBW than LBW pigs on the R regime but similar in LBW and HBW pigs on the A regime. Gondret et al. (2005), Paredes et al. (2014), and Van der Peet-Schwering et al. (2020) also observed lower IGF-1 concentrations in LWB pigs. It is known that prenatal muscle development and postnatal muscle growth are controlled by the IGF system (Oksbjerg et al., 2004) and that a high ADG in HBW pigs is associated with a higher IGF-1 plasma concentration (Paredes et al., 2014). A higher plasma IGF-1 concentration is also associated with a higher feed intake (Oksbjerg et al., 2004), a higher dietary protein level, and an adequate dietary supply of all essential AA (Thissen et al., 1994; Sánchez-Gómez et al., 1999). In both children and adults, it was shown that circulating IGF-1 is markedly influenced by dietary nutrient supply, falling during starvation and rising rapidly upon refeeding (Leger et al., 1996). Maybe, LBW pigs are more sensitive to a restricted protein supply than HBW pigs resulting in a lower plasma IGF-1 concentration on the R regime, whereas on the A regime both LBW and HBW pigs receive sufficient nutrients, including AA relative to their requirements for near maximum performance, resulting in similar plasma IGF-1 concentrations.

### Breeding value for protein deposition

On the A regime, N retention in the LBV and HBV pigs was 1.104 and 1.214 g/(kg BW^0.75^ · d), respectively, equivalent to a protein deposition of 6.90 and 7.59 g/(kg BW^0.75^ · d), respectively, or 163 and 177 g/d, respectively (Supplementary Table S2). The higher N retention (in g/d and in g/[kg BW^0.75^ · d]) resulted in a higher N efficiency of about 5% (55.2% vs. 50.2% based on total N intake and 60.0% vs. 54.4% based on fecal digestible N intake) in the HBV pigs compared with the LBV pigs on the A regime. In pigs on the R regime, however, N retention was similar in LBV and HBV pigs (131 and 132 g/d, respectively) resulting in a similar N efficiency (%). A restricted supply of dietary protein and AA in pigs on the R regime probably explains the similar N retention and N efficiency (%) in LBV and HBV pigs. As far as we know, there are no other studies in which the N efficiency (%) in LBV and HBV growing pigs was measured. Van der Peet-Schwering et al. (2013), however, measured the feed efficiency in GF pigs with a high or low EBV for daily gain. Pigs with a high EBV for daily gain had a higher feed efficiency, which is in line with the higher N efficiency in pigs with a high EBV for protein deposition.

Total tract digestibility of DM, N, and GE did not differ between LBV and HBV growing pigs. These results are in accordance with our expectations. A difference in nutrient digestibility between the LBV and HBV pigs was not expected because the pigs were selected for a difference in genetic capacity in protein deposition and not for a difference in digestion capacity. As far as we know, there are no other studies in which the total tract digestibility of DM, N, and GE in LBV and HBV growing pigs was measured.

The concentration of IGF-1 in blood plasma was similar in LBV and HBV pigs on the R regime but higher in HBV than LBV pigs on the A regime. Clutter et al. (1995) studied the plasma IGF-1 concentration in gilts from lines of pigs selected for either fast or slow postweaning ADG for seven generations during a period of feed deprivation and during refeeding. The fast-growing gilts had greater concentrations of plasma IGF-1 than slow-growing gilts during both feed deprivation (217 vs. 145 ng/mL) and during refeeding (165 vs. 128 ng/mL). Plasma IGF-1 concentration decreased during feed deprivation and increased during refeeding. In the study of Clutter et al. (1995) and in our study, the effect of feeding regime on plasma IGF-1 concentration was greater in pigs with a high EBV or high ADG than in pigs with a low EBV or low ADG. Maybe, pigs with a high EBV are more sensitive to restrictions in dietary nutrient supply including protein and AA than pigs with a low EBV for protein deposition.

The concentration of creatinine in blood plasma was higher in HBV than LBV pigs. The metabolomic analysis of the same samples confirmed this observation. Plasma and urinary creatinine concentration is a reflection of creatine phosphate degradation in muscle tissue and its excretion via the kidneys into the urine. The amount of creatinine removed is proportional to total creatine and creatine phosphate in the body and consequently also to the total muscle mass of muscle in pigs (Janicki and Buzala, 2013). Although the HBV pigs had higher circulating and urinary levels of creatinine compared with LBV pigs, the BW of pigs in both groups during the study was similar. We did not measure body protein content, but the results on N efficiency indicate that HBV pigs deposited more protein in the body than LBV pigs, which is in line with the contrast in plasma creatinine levels between both groups.

Metabolomics analysis revealed that the plasma concentrations of a hexose, most likely glucose, and phenylalanine and methionine, both essential AA, were higher in HBV pigs. In addition, four LPC contributed to the separation between HBV and LBV pigs. Lower circulating levels of essential AA were observed in stunned children (Semba et al., 2016), whereas protein restriction and hypercholesterolemia in mice were related to lower concentrations of circulating essential AA and intrauterine growth restriction (Bhasin et al., 2009). Stunning was also related to altered LPC patterns in blood plasma in children. The LPC that contributed to the separation of groups of pigs in the present study were lower in stunted children in the study of Semba et al. (2016) compared with a reference group of children. The LPC are molecules derived from phosphatidylcholines and are part of cell membranes and the monolayer of lipoproteins. To absorb biliary phosphatidylcholines across the intestinal brush border membrane, phosphatidylcholines are hydrolyzed to LPC and fatty acids, where LPC might play a role in chylomicron formation (Van der Veen et al., 2017). In humans, high circulating levels of LPC are related to cardiovascular and neurodegenerative diseases (Law et al., 2019). Hence, the higher levels of circulating AA and changed LPC composition in HBV compared with LBV pigs in the present study might be related to a higher body protein deposition.

### Dietary protein supply

Restricting the dietary protein supply by 30% reduced the N retention by 22% (in g/d and in g/[kg BW^0.75^ · d]) and N digestibility by 3.2%. Similar results were reported by Kampman-Van de Hoek et al. (2015) and Van der Peet-Schwering et al. (2020). In their studies, N retention was 20% and 30% lower in pigs on the R regime compared with the A regime, and the total tract N digestibility was 2% and 3% lower. The lower N retention on the R regime is due to the lower intake of protein and essential AA. The lower apparent total tract N digestibility is likely due to a proportionally greater excretion of basal endogenous N in pigs on the R regime, as the relative contribution of endogenous N to total fecal N excretion decreases with increasing dietary protein supply (Fan et al., 1994). Restricting the dietary protein supply increased N efficiency (%). Similar results were reported by Kampman-Van de Hoek et al. (2015). They also reported a higher N efficiency (%) in pigs on a similar R regime. In our study and the one of Kampman-Van de Hoek et al. (2015), N intake was reduced by about 25% to 30%, whereas urinary N excretion was reduced by about 35% to 38% in pigs on the R regime resulting in a higher N efficiency (%) in pigs fed a restricted amount of protein. The concentrations of N and urea in the urine also were reduced by about 38% in pigs on the R regime compared with the A regime. The concentration of urea in urine is a reflection of urinary N excretion as a major part of the N from AA after oxidation is transformed into urea in the liver and excreted via the urine.

The concentration of IGF-1 in blood plasma was higher in pigs on the A regime than in pigs on the R regime as in the study of Van der Peet-Schwering et al. (2020). This was expected as a high dietary protein level and supplementation of protein-restricted diets with essential AA are associated with increased total IGF-1 (Thissen et al., 1994; Sánchez-Gómez et al., 1999).

Restricting dietary protein supply reduced the concentration of urea in blood plasma but it did not change the plasma concentration of α-amino N as an indicator of the free AA pool in plasma. This confirms the homeostatic regulation of concentrations of essential nutrients in the blood plasma of pigs in which body protein turnover plays an important role.

### Conclusions

In conclusion, N efficiency and absolute N retention are higher in HBV than in LBV pigs on protein-adequate diets, indicating that breeding pigs for a high protein deposition capacity may support the further increase of sustainability of pig production. Plasma concentrations of creatinine, AA, and other metabolites in plasma and urine, as determined by metabolomics approaches, can be potential indicators for the genetic capacity for protein deposition and for the dietary protein and AA supply in pigs. In future precision feeding concepts aiming to further optimize protein and AA efficiency in pigs, the variation in genetic capacity for protein deposition should be considered as a factor determining nutrient requirements, growth performance, and N efficiency of pigs.

## Supplementary Material

skab101_suppl_Supplementary_Materials

## Data Availability

The metabolomics data are uploaded to the Metabolomics Workbench (Sud et al., 2016): http://dx.doi.org/10.21228/M80M6C.

## Abbreviations

A: protein adequate
AA: amino acids
AID: apparent ileal digestible
ATTD: apparent total tract digestibility
BiW: birth weight
BW: body weight
BW^0.75^: metabolic body weight
DM: dry matter
EBV: estimated breeding value
GE: gross energy
GF: growing and finishing
HBV: high estimated breeding value
HBW: high birth weight
IGF-1: insulin-like growth factor
LBV: low estimated breeding value
LBW: low birth weight
LPC: lysophosphatidylcholines
ME: metabolizable energy
NE: net energy
PC: principal component
PCA: principal component analysis
PLS-DA: partial least square discriminant analysis
QC: quality control
R: protein restricted
RT: retention time
VIP: variable importance in projection
