# Supplementary material for: The effects of birth weight and estimated breeding value for protein deposition on nitrogen efficiency in growing pigs
Source: J Anim Sci. 2021 Mar 29;99(6):skab101. doi: 10.1093/jas/skab101 (PMC8188818; doi:10.1093/jas/skab101)
Supplement: skab101_suppl_Supplementary_Materials [file skab101_suppl_supplementary_materials.zip › skab101_suppl_Supplementary_Tables.docx]

Supplementary Table S1: Metabolites identified as contributing to the discrimination in the partial least squares discriminant analysis models

| **Metabolite** | **Adduct** | **Mode^1^** | **RT^2^** | **MZ** | **Level of ID^3^** | **Model^4^** | **Kegg /HMDB** | **Pathway** |
| --- | --- | --- | --- | --- | --- | --- | --- | --- |
| *Plasma* |  |  |  |  |  |  |  |  |
| Sulfate | [Fragment] | Neg | 0.97 | 78.959 | 2 | D | C00059 | Sulfur metabolism |
| Sulfate | [M-H]- | Neg | 0.97 | 96.969 | 2 | D | C00059 | Sulfur metabolism |
| Lactic acid | [M-H]- | Neg | 0.97 | 89.024 | 1 | D | C00186 | Glycolysis/Gluconeogenesis |
| Urea | [M+H]+ | Pos | 0.71 | 61.040 | 1 | D | C00086 | Purine, pyrimidine, and arginine and proline metabolism |
| Choline | [M+H]+ | Pos | 0.66 | 104.107 | 1 | EBV | C00114 | Glycine, serine and threonine metabolism |
| Creatinine | [M+H]+ | Pos | 0.70 | 114.066 | 1 | EBV/D | C00791 | Arginine and proline metabolism |
| Creatine | [M+H]+ | Pos | 0.71 | 132.077 | 1 | EBV/D | C00300 | Glycine, serine and threonine metabolism |
| Betaine | [M]+ | Pos | 0.80 | 118.086 | 1 | D | C00719 | Glycine, serine and threonine metabolism |
| 2-Piperidinone | [M+H]+ | Pos | 2.04 | 100.076 | 1 | EBV | HMDB11749 | Derivative of piperidine |
| C6H12O6 | [2M+Na]+ | Pos | 0.70 | 383.117 | 3 | EBV |  |  |
| C6H12O6 | [M+Na]+ | Pos | 0.70 | 203.053 | 3 | EBV |  |  |
| Proline | [M+H]+ | Pos | 0.73 | 116.071 | 1 | EBV/D | C00148 | Arginine and proline metabolism |
| Proline | [Fragment] | Pos | 0.73 | 70.065 | 1 | EBV | C00148 | Arginine and proline metabolism |
| Valine | [M+Na]+ | Pos | 0.82 | 130.087 | 1 | EBV/D | C00183 | Valine, leucine and isoleucine degradation |
| Methionine | [M+H]+ | Pos | 0.94 | 150.059 | 1 | EBV | C00073 | Cysteine and methionine metabolism |
| Tyrosine | [M+H]+ | Pos | 1.14 | 182.082 | 1 | EBV/D | C00082 | Tyrosine metabolism |
| Tyrosine | [Fragment] | Pos | 1.14 | 136.076 | 1 | EBV | C00082 | Tyrosine metabolism |
| Tyrosine | [Fragment] | Pos | 1.14 | 165.055 | 1 | EBV/D | C00082 | Tyrosine metabolism |
| Tyrosine | [2M+Na]- | Neg | 1.15 | 383.122 | 1 | D | C00082 | Tyrosine metabolism |
| Tyrosine | [M-H]- | Neg | 1.16 | 180.067 | 1 | D | C00082 | Tyrosine metabolism |
| Leucine | [Fragment] | Pos | 1.23 | 86.097 | 1 | EBV/D | C00123 | Valine, leucine and isoleucine degradation |
| Leucine | [M+H]+ | Pos | 1.25 | 132.102 | 1 | EBV/D | C00123 | Valine, leucine and isoleucine degradation |
| Leucine | [M-H]- | Neg | 1.27 | 130.087 | 1 | D | C00407 | Valine, leucine and isoleucine degradation/biosynthesis |
| Phenylalanine | [M+H]+ | Pos | 2.11 | 166.087 | 1 | EBV/D | C00079 | Phenylalanine metabolism |
| Phenylalanine | [Fragment] | Pos | 2.11 | 120.081 | 1 | EBV/D | C00079 | Phenylalanine metabolism |
| Phenylalanine | [Fragment] | Pos | 2.11 | 103.055 | 1 | EBV | C00079 | Phenylalanine metabolism |
| Phenylalanine | [M-H]- | Neg | 2.11 | 164.072 | 1 | D | C00079 | Phenylalanine metabolism |
| 4-Trimethylammonio-butanoic acid | [Fragment] | Pos | 2.57 | 100.112 | 2 | D | C01181 | Lysine degradation |
| 4-Trimethylammonio-butanoic acid | [M+H]+ | Pos | 2.57 | 146.118 | 2 | D | C01181 | Lysine degradation |
| Tryptophan | [Fragment] | Pos | 2.87 | 188.071 | 1 | D | C00078 | Tryptophan metabolism |
| Tryptophan | [M-H]- | Neg | 2.88 | 203.083 | 1 | D | C00078 | Tryptophan metabolism |
| Hydroxyphenyllactic acid | [M-H]- | Neg | 2.92 | 181.051 | 1 | D | C03672 | Tyrosine metabolism |
| Ketoisoleucine | [M-H]- | Neg | 3.21 | 129.056 | 1 | D | C00671 | Valine, leucine and isoleucine degradation |
| Ketoleucine | [M-H]- | Neg | 3.54 | 129.056 | 1 | D | C00233 | Valine, leucine and isoleucine degradation |
| Phenylacetylglycine | [M-H]- | Neg | 3.90 | 192.067 | 1 | D | C05598 | Phenylalanine metabolism |
| Indoxylsulfuric acid | [M-H]- | Neg | 3.54 | 212.002 | 1 | D |  | Microbial metabolite |
| p-Cresol glucuronide | [M-H]- | Neg | 4.06 | 283.083 | 2 | D |  | Microbial metabolite |
| p-Cresol sulfate | [M-H]- | Neg | 4.11 | 187.007 | 1 | D |  | Microbial metabolite |
| Indolelactic acid | [M-H]- | Neg | 4.61 | 204.067 | 2 | D | C02043 | Microbial metabolite |
| 2-Hydroxybutyric acid | [M-H]- | Neg | 1.56 | 103.040 | 1 | D | C05984 | Propanoate metabolism |
| Azelaic acid | [M-H]- | Neg | 4.90 | 187.098 | 1 | D | C08261 | Fatty acids and conjugates metabolism |
| Taurodeoxycholic acid | [M-H]- | Neg | 5.87 | 498.290 | 2 | D | C05463 | Bile acid metabolism |
| Glycoursodeoxycholic acid /Glycodeoxycholate | [M-H]- | Neg | 6.66 | 448.307 | 2 | D |  | Bile acid metabolism |
| Bile acid | [M+H]+ | Pos | 7.65 | 450.322 | 3 | EBV |  | Bile acid metabolism |
| Bile acid | [M+H]+ | Pos | 10.85 | 393.299 | 3 | EBV |  | Bile acid metabolism |
| PC(18:2/0) | [M+FA-H]- | Neg | 9.19 | 564.331 | 2 | D |  | Glycerophospholipid metabolism |
| PC(18:2/0) | [M+H]+ | Pos | 9.21 | 520.341 | 2 | EBV |  | Glycerophospholipid metabolism |
| PC(18:2/0) | [M+Na]+ | Pos | 9.21 | 542.323 | 2 | EBV |  | Glycerophospholipid metabolism |
| PC(18:2/0) | [M+H]+ | Pos | 9.21 | 520.341 | 2 | D |  | Glycerophospholipid metabolism |
| PC(16:0/0) | [M+FA-H]- | Neg | 9.62 | 540.331 | 2 | D |  | Glycerophospholipid metabolism |
| PC(16:0/0) | [M+H]+ | Pos | 9.64 | 496.341 | 3 | EBV/D |  | Glycerophospholipid metabolism |
| PC(18:1/0) | [M+FA-H]- | Neg | 9.95 | 566.346 | 2 | D |  | Glycerophospholipid metabolism |
| PC(18:1/0) | [M+H]+ | Pos | 9.98 | 522.357 | 2 | EBV/D |  | Glycerophospholipid metabolism |
| PC(18:0/0) | [M+H]+ | Pos | 10.96 | 524.372 | 3 | EBV |  | Glycerophospholipid metabolism |
| PC(18:0/0) | [M+Na]+ | Pos | 10.96 | 546.354 | 3 | EBV |  | Glycerophospholipid metabolism |
| Sulfated compound | [M-H]- | Neg | 4.59 | 226.018 | 3 | D |  | Sulfate conjugation |
| Unidentified | [M-H]- | Neg | 0.71 | 215.033 | 4 | D |  |  |
| Unidentified | [M+H]+ | Pos | 0.75 | 229.119 | 4 | EBV |  |  |
| Unidentified | [M-H]- | Neg | 1.17 | 189.041 | 4 | D |  |  |
| Unidentified | [M+H]+ | Pos | 4.58 | 239.090 | 4 | EBV/D |  |  |
| Unidentified | [M+H]+ | Pos | 8.38 | 398.243 | 4 | D |  |  |
| *Urine* |  |  |  |  |  |  |  |  |
| Sulfate | [M-H]- | Neg | 0.63 | 96.9600 | 2 | D | C00059 | Sulfur metabolism |
| Taurine | [M-H]- | Neg | 0.68 | 124.0073 | 1 | D | C00245 | Sulfur metabolism |
| Creatinine | [2M+Na]+ | Pos | 0.70 | 249.1072 | 1 | EBV | C00791 | Arginine and proline metabolism |
| Creatinine | [M+Na]+ | Pos | 0.70 | 136.0481 | 1 | EBV | C00791 | Arginine and proline metabolism |
| Creatinine | [M+K]+ | Pos | 0.71 | 152.0220 | 1 | D | C00791 | Arginine and proline metabolism |
| Creatinine | [M+K]+ | Pos | 0.71 | 152.0220 | 1 | EBV | C00791 | Arginine and proline metabolism |
| Creatinine | [M+H]+ | Pos | 0.72 | 114.0661 | 1 | EBV/D | C00791 | Arginine and proline metabolism |
| Piperidine | [M+H]+ | Pos | 0.83 | 86.0965 | 1 | EBV | C01746 | Protein digestion and absorption |
| 2-Piperidinone | [M+H]+ | Pos | 2.02 | 100.0758 | 1 | EBV/D | HMDB11749 | Derivative of piperidine |
| C5H9NO | [M+H]+ | Pos | 2.22 | 100.0759 | 3 | EBV |  |  |
| Citric acid | [M+H]+ | Pos | 0.94 | 193.0344 | 1 | D | C00158 | Citrate cycle (TCA cycle)/Alanine, aspartate and glutamate metabolism |
| Citric acid | [M+Na]+ | Pos | 0.93 | 215.0163 | 1 | EBV | C00158 | Citrate cycle (TCA cycle)/Alanine, aspartate and glutamate metabolism |
| Citric acid | [M-H]- | Neg | 0.94 | 191.0196 | 1 | EBV/D | C00158 | Citrate cycle (TCA cycle)/Alanine, aspartate and glutamate metabolism |
| Citric acid | [Fragment] | Neg | 0.94 | 111.0087 | 1 | EBV/D | C00158 | Citrate cycle (TCA cycle)/Alanine, aspartate and glutamate metabolism |
| Succinic acid | [M-H]- | Neg | 1.23 | 117.0194 | 1 | D | C00042 | Citrate cycle (TCA cycle) |
| 3-hydroxy-3-methyl-Glutaric acid | [M-H]- | Neg | 1.31 | 161.0456 | 1 | D | C03761 | Leucine metabolism |
| Hydroxyphenyllactic acid | [M-H]- | Neg | 2.89 | 181.0506 | 1 | D | C03672 | Tyrosine metabolism |
| Phenylacetylglycine | [M-H]- | Neg | 3.85 | 192.0662 | 1 | EBV/D | C05598 | Phenylalanine metabolism |
| Phenylacetylglycine | [Fragment] | Pos | 3.86 | 76.0393 | 1 | EBV/D | C05598 | Phenylalanine metabolism |
| Phenylacetylglycine | [M+H]+ | Pos | 3.86 | 194.0812 | 1 | EBV/D | C05598 | Phenylalanine metabolism |
| Phenylacetylglycine | [Fragment] | Pos | 3.86 | 91.0542 | 1 | EBV/D | C05598 | Phenylalanine metabolism |
| L-Formylkynurenine | [M+H-H2O]+ | Pos | 3.93 | 219.0767 | 2 | EBV | C02700 | Tryptophan metabolism |
| Picolinoylglycine | [M+H]+ | Pos | 2.98 | 181.0610 | 2 | D | HMDB0059766 | N-acyl-alpha amino acid |
| L-Formylkynurenine | [Fragment] | Pos | 3.93 | 144.0445 |  | EBV | C02700 | Tryptophan metabolism |
| Acetyl-DL-Leucine | [M-H]- | Neg | 3.75 | 172.0979 | 1 | D | C02710 | Leucine and derivatives |
| Hippuric acid sulfate | [M-H]- | Neg | 2.32 | 258.0078 | 2 | EBV/D |  | Microbial metabolite |
| Indoxylsulfuric acid | [M-H]- | Neg | 3.46 | 212.0021 | 1 | EBV/D | HMDB0000682 | Microbial metabolite |
| 3-hydroxyhippuric acid | [M-H]- | Neg | 2.85 | 194.0459 | 2 | D | HMDB0006116 | Microbial metabolite |
| Hippuric acid | [Fragment] | Pos | 3.50 | 77.0386 | 1 | EBV | C01586 | Microbial metabolite |
| Hippuric acid | [Fragment] | Pos | 3.50 | 105.0335 | 1 | EBV/D | C01586 | Microbial metabolite |
| Hippuric acid | [M+H]+ | Pos | 3.50 | 180.0657 | 1 | EBV/D | C01586 | Microbial metabolite |
| Hippuric acid | [M-H]- | Neg | 3.51 | 178.0507 | 1 | EBV/D | C01586 | Microbial metabolite |
| p-Cresol glucuronide | [2M-H]- | Neg | 4.00 | 567.1711 | 2 | EBV/D | HMDB0011686 | Microbial metabolite |
| p-Cresol glucuronide | [M-H]- | Neg | 4.01 | 283.0818 | 2 | EBV/D | HMDB0011686 | Microbial metabolite |
| p-Cresol sulfate | [M-H]- | Neg | 4.04 | 187.0067 | 1 | EBV/D | HMDB0011635 | Microbial metabolite |
| N-Acetyl-DL-tryptophan | [M+H]+ | Pos | 4.51 | 247.1080 | 1 | EBV | HMDB0013713 | Microbial metabolite |
| Cinnamoylglycine | [M-H]- | Neg | 4.76 | 204.0665 | 1 | EBV | HMDB0011621 | Microbial metabolite |
| Cinnamoylglycine | [Fragment] | Pos | 4.77 | 131.0493 | 1 | EBV | HMDB0011621 | Microbial metabolite |
| Cinnamoylglycine | [M+H]+ | Pos | 4.77 | 206.0815 | 1 | EBV | HMDB0011621 | Microbial metabolite |
| Oxindole | [M+H]+ | Pos | 3.43 | 134.0602 | 2 | D | C12312 | Microbial metabolite |
| 2-Methylbutyrylglycine /Valerylglycine | [M-H]- | Neg | 3.00 | 158.0822 | 2 | EBV/D |  | Acylglycine, mitochondrial fatty-acid beta-oxidation |
| 2-Methylbutyrylglycine /Valerylglycine | [M+H]+ | Pos | 3.00 | 160.0970 | 2 | EBV/D |  | Acylglycine, mitochondrial fatty-acid beta-oxidation |
| 2-Methylbutyrylglycine /Valerylglycine | [Fragment] | Pos | 3.00 | 76.0394 | 2 | EBV |  | Acylglycine, mitochondrial fatty-acid beta-oxidation |
| Azelaic acid | [M-H]- | Neg | 4.83 | 187.0974 | 1 | EBV/D | C08261 | Fatty acids and conjugates metabolism |
| Dodecanedioic acid | [M-H]- | Neg | 6.71 | 229.1445 | 1 | EBV | C02678 | Intermediate in the pathways of lipids and carbohydrates |
| 7-Methylguanine | [M+H]+ | Pos | 0.90 | 166.0724 | 2 | EBV/D | C02242 | A metabolite of DNA methylation and depurination |
| Niazirin | [M+H]+ | Pos | 3.63 | 280.1218 | 2 | EBV/D | HMDB32807 | Phenolic glycoside |
| Heliotrine | [M+H]+ | Pos | 4.92 | 314.1968 | 2 | EBV |  | Alkaloid derived from ornithine |
| 6-Hydroxy-5-methoxy-indole glucuronide | [Fragment] | Pos | 3.12 | 146.0602 | 2 | EBV |  | Metabolism of xenobiotics |
| 6-Hydroxy-5-methoxy-indole glucuronide | [M+H]+ | Pos | 3.12 | 340.1030 | 2 | EBV |  | Metabolism of xenobiotics |
| Acetaminophen glucuronide | [M+H]+ | Pos | 3.60 | 328.1030 | 2 | EBV/D |  | Metabolism of xenobiotics |
| 6-Hydroxy-5-methoxy-indole glucuronide | [M+H]+ | Pos | 3.60 | 340.1030 | 2 | EBV |  | Metabolism of xenobiotics |
| Melatonin glucuronide | [M+H]+ | Pos | 3.82 | 409.1601 | 2 | EBV |  | Metabolism of xenobiotics |
| Glucoronidated compound | [M-H]- | Neg | 3.13 | 338.0879 | 3 | EBV |  | Metabolism of xenobiotics |
| Glucoronidated compound | [M-H]- | Neg | 3.33 | 322.0931 | 3 | D |  | Metabolism of xenobiotics |
| Glucoronidated compound | [M+H]+ | Pos | 4.00 | 302.1238 | 3 | EBV/D |  | Metabolism of xenobiotics |
| Glucoronidated compound | [M-H]- | Neg | 4.26 | 449.2024 | 3 | EBV/D |  | Metabolism of xenobiotics |
| Glucoronidated compound | [M-H]- | Neg | 4.73 | 277.1292 | 3 | EBV |  | Metabolism of xenobiotics |
| Glucoronidated compound | [M-H]- | Neg | 4.97 | 431.1920 | 3 | EBV/D |  | Metabolism of xenobiotics |
| Glucoronidated compound | [M-H]- | Neg | 5.26 | 433.2077 | 3 | EBV |  | Metabolism of xenobiotics |
| Glucoronidated compound | [M-H]- | Neg | 5.42 | 387.1659 | 3 | EBV |  | Metabolism of xenobiotics |
| Glucoronidated compound | [M-H]- | Neg | 5.55 | 505.2652 | 3 | EBV |  | Metabolism of xenobiotics |
| Glucoronidated compound | [M-H]- | Neg | 5.59 | 433.2077 | 3 | D |  | Metabolism of xenobiotics |
| Glucoronidated compound | [M-H]- | Neg | 5.81 | 415.1972 | 3 | EBV/D |  | Metabolism of xenobiotics |
| Glucoronidated compound | [M-H]- | Neg | 6.12 | 461.2391 | 3 | EBV/D |  | Metabolism of xenobiotics |
| Glucoronidated compound | [M-H]- | Neg | 6.26 | 417.2128 | 3 | EBV |  | Metabolism of xenobiotics |
| 3-Hydroxyanthranilic acid sulfate | [M-H]- | Neg | 2.92 | 231.9920 | 2 | D |  | Sulfate conjugation |
| Sulfated steroid | [M-H]- | Neg | 5.90 | 367.1582 | 3 | EBV/D |  | Sulfate conjugation |
| Sulfated steroid | [M-H]- | Neg | 6.24 | 367.1584 | 3 | EBV/D |  | Sulfate conjugation |
| Sulfated steroid | [M-H]- | Neg | 6.40 | 369.1740 | 3 | EBV/D |  | Sulfate conjugation |
| Sulfated steroid | [M-H]- | Neg | 6.62 | 369.1741 | 3 | EBV |  | Sulfate conjugation |
| Sulfated compound | [M-H]- | Neg | 2.68 | 242.0128 | 3 | EBV/D |  | Sulfate conjugation |
| Sulfated compound | [M-H]- | Neg | 3.57 | 167.0382 | 3 | EBV/D |  | Sulfate conjugation |
| Sulfated compound | [M-H]- | Neg | 3.70 | 230.0127 | 3 | EBV/D |  | Sulfate conjugation |
| Sulfated compound | [M-H]- | Neg | 4.32 | 179.0382 | 3 | EBV |  | Sulfate conjugation |
| Sulfated compound | [M-H]- | Neg | 4.52 | 226.0177 | 3 | EBV/D |  | Sulfate conjugation |
| Sulfated compound | [M-H]- | Neg | 4.58 | 181.0539 | 3 | EBV/D |  | Sulfate conjugation |
| Sulfated compound | [M-H]- | Neg | 4.86 | 381.1375 | 3 | EBV |  | Sulfate conjugation |
| Sulfated compound | [M-H]- | Neg | 5.12 | 226.0178 | 3 | EBV/D |  | Sulfate conjugation |
| Sulfated compound | [M-H]- | Neg | 5.61 | 303.1274 | 3 | D |  | Sulfate conjugation |
| Unidentified | [M+H]+ | Pos | 0.94 | 210.0609 | 4 | D |  |  |
| Unidentified | [M+H]+ | Pos | 0.94 | 175.0238 | 4 | D |  |  |
| Unidentified | [M+H]+ | Pos | 0.94 | 210.0609 | 4 | EBV |  |  |
| Unidentified | [M+H]+ | Pos | 0.97 | 202.1189 | 4 | EBV |  |  |
| Unidentified | [M-H]- | Neg | 1.06 | 129.0193 | 4 | D |  |  |
| Unidentified | [M+H]+ | Pos | 1.14 | 245.1610 | 4 | EBV |  |  |
| Unidentified | [M+H]+ | Pos | 1.19 | 174.1240 | 4 | EBV |  |  |
| Unidentified | [M+H]+ | Pos | 1.36 | 326.0875 | 4 | EBV |  |  |
| Unidentified | [M+H]+ | Pos | 1.56 | 144.0658 | 4 | D |  |  |
| Unidentified | [M+H]+ | Pos | 1.88 | 245.1501 | 4 | EBV/D |  |  |
| Unidentified | [M+H]+ | Pos | 1.92 | 136.0396 | 4 | D |  |  |
| Unidentified | [M+H]+ | Pos | 2.48 | 279.1344 | 4 | EBV/D |  |  |
| Unidentified | [M+H]+ | Pos | 3.11 | 357.1297 | 4 | EBV |  |  |
| Unidentified | [M+H]+ | Pos | 3.17 | 288.1810 | 4 | D |  |  |
| Unidentified | [M+H]+ | Pos | 3.21 | 260.1497 | 4 | EBV |  |  |
| Unidentified | [M+H]+ | Pos | 4.20 | 233.0924 | 4 | EBV/D |  |  |
| Unidentified | [Fragment] | Pos | 4.22 | 130.0653 | 4 | EBV |  |  |
| Unidentified | [M-H]- | Neg | 4.45 | 380.1746 | 4 | D |  |  |
| Unidentified | [M+H]+ | Pos | 4.96 | 510.2715 | 4 | EBV |  |  |
| Unidentified | [M+H]+ | Pos | 5.27 | 432.3478 | 4 | D |  |  |
| Unidentified | [M-H]- | Neg | 5.28 | 309.1342 | 4 | D |  |  |
| Unidentified | [M+H]+ | Pos | 5.34 | 444.3116 | 4 | EBV |  |  |
| Unidentified | [M+H]+ | Pos | 5.37 | 130.0653 | 4 | EBV/D |  |  |
| Unidentified | [M-H]- | Neg | 5.42 | 291.1448 | 4 | EBV |  |  |
| Unidentified | [M+H]+ | Pos | 5.95 | 307.2021 | 4 | D |  |  |
| Unidentified | [M+H]+ | Pos | 5.95 | 635.3787 | 4 | EBV |  |  |
| Unidentified | [M+H]+ | Pos | 5.95 | 307.2021 | 4 | EBV |  |  |

^1^Pos, positive; Neg, negative.

^2^Retention time.

^3^Level of identification: Identified metabolites (level 1), putatively annotated compounds (level 2), putatively characterized compound classes (level 3), and unknown compounds (level 4).

^4^Partial least squares discriminant analysis model where the metabolites was identified as discriminating (D: Diet, EBV: Estimated breeding value for protein deposition)

Supplementary Table S2: Effects of birth weight (BiW), estimated breeding value for protein deposition (EBV), and dietary protein supply on nitrogen (N) balance parameters (g/d) in male growing pigs

|  | Birth weight^1^ | |  | EBV^1^ | |  | Dietary protein supply^1^ | |  | *P*-value | | |
| --- | --- | --- | --- | --- | --- | --- | --- | --- | --- | --- | --- | --- |
|  | Low | High | SEM | Low | High | SEM | Adequate | Restricted | SEM | BiW | BV | Diet |
| N intake | 41.68 | 47.56 | 0.62 | 45.02 | 44.22 | 0.62 | 51.55 | 37.69 | 0.19 | <0.001 | 0.37 | <0.001 |
| Fecal N | 3.74 | 3.95 | 0.13 | 3.93 | 3.75 | 0.13 | 3.73 | 3.95 | 0.10 | 0.27 | 0.33 | 0.12 |
| Urinary N^2^ | 15.16 | 18.14 | 0.50 | 17.54 | 15.76 | 0.50 | 20.65 | 12.65 | 0.35 | <0.001 | 0.02 | <0.001 |
| N retention^3^ | 22.78 | 25.48 | 0.61 | 23.55 | 24.71 | 0.61 | 27.17 | 21.09 | 0.38 | 0.004 | 0.19 | <0.001 |
| N efficiency^4,5^, % | 54.8 | 53.7 | 1.03 | 52.7 | 55.8 | 1.03 | 52.7 | 55.8 | 0.67 | 0.42 | 0.04 | 0.003 |

^1^ Number of observations: 36 and 37 in low and high birth weight pigs, respectively; 36 and 37 in low and high EBV pigs, respectively; 36 and 37 on the adequate and restricted protein regime, respectively.

^2^ An EBV × Diet interaction was observed (*P* = 0.009): A regime: urinary N in pigs with low and high EBV is 22.23 and 19.08 g/d, respectively; R regime: urinary N in pigs with low and high EBV is 12.85 and 12.45 g/d, respectively.

^3^ An EBV × Diet interaction was observed (*P* = 0.06): A regime: N retention in pigs with low and high EBV is 26.07 and 28.26 g/d, respectively; R regime: N retention in pigs with low and high EBV is 21.03 and 21.15 g/d, respectively.

^4^ N efficiency = 100% x N retention / N intake

^5^ An EBV × Diet interaction was observed (*P* = 0.04): A regime: N efficiency in pigs with low and high EBV is 50.2 and 55.2%, respectively; R regime: N efficiency in pigs with low and high EBV is 55.2 and 56.3%, respectively.

Supplementary Figure S1. Principle component plot of plasma samples in positive mode (A), plasma samples in negative mode (B), urine samples in positive mode (C), and urine samples in negative mode (D). Colours indicate the different dietary treatments (Adequate and Restricted) imposed to the pigs.
