# Supplementary figures and images for: The effects of birth weight and estimated breeding value for protein deposition on nitrogen efficiency in growing pigs
Source: J Anim Sci. 2021 Mar 29;99(6):skab101. doi: 10.1093/jas/skab101 (PMC8188818; doi:10.1093/jas/skab101)

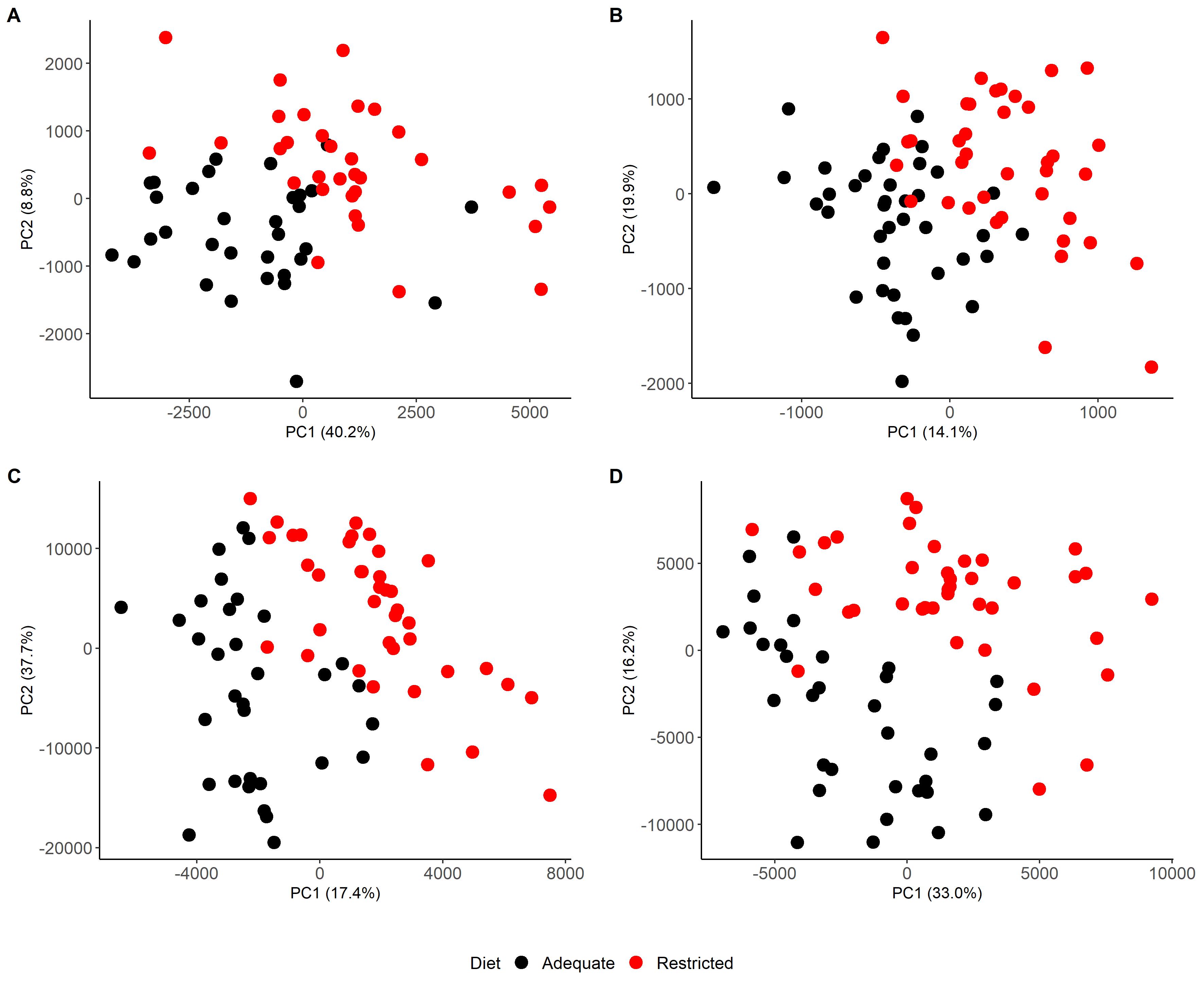

Supplement: skab101_suppl_Supplementary_Materials [file skab101_suppl_supplementary_materials.zip › skab101_suppl_Supplementary_Figure_S1.jpeg]
